# Supplementary material for: A fault-intrusion-tolerant system and deadline-aware algorithm for scheduling scientific workflow in the cloud
Source: PeerJ Comput Sci. 2021 Nov 2;7:e747. doi: 10.7717/peerj-cs.747 (PMC8576554; doi:10.7717/peerj-cs.747)
Supplement: Supplemental Information 6 [file peerj-cs-07-747-s006.zip › Myproject1.1/docs/index-files/index-14.html]

O-Index


JavaScript is disabled on your browser.


- Overview
- Package
- Class
- Use
- Tree
- Deprecated
- Index
- Help

- Prev Letter
- Next Letter

- Frames
- No Frames

- All Classes

A B C D E F G H I J L M N O P Q R S T U V W Z 


## O

optimizeAllocation(List<? extends Vm>) - Method in class org.cloudbus.cloudsim.network.datacenter.NetworkVmAllocationPolicy


optimizeAllocation(List<? extends Vm>) - Method in class org.cloudbus.cloudsim.power.PowerVmAllocationPolicyMigrationAbstract
:   Optimize allocation of the VMs according to current utilization.

optimizeAllocation(List<? extends Vm>) - Method in class org.cloudbus.cloudsim.power.PowerVmAllocationPolicySimple


optimizeAllocation(List<? extends Vm>) - Method in class org.cloudbus.cloudsim.VmAllocationPolicy
:   Optimize allocation of the VMs according to current utilization.

optimizeAllocation(List<? extends Vm>) - Method in class org.cloudbus.cloudsim.VmAllocationPolicySimple


org.cloudbus.cloudsim - package org.cloudbus.cloudsim


org.cloudbus.cloudsim.core - package org.cloudbus.cloudsim.core


org.cloudbus.cloudsim.core.predicates - package org.cloudbus.cloudsim.core.predicates


org.cloudbus.cloudsim.distributions - package org.cloudbus.cloudsim.distributions


org.cloudbus.cloudsim.examples - package org.cloudbus.cloudsim.examples


org.cloudbus.cloudsim.examples.network - package org.cloudbus.cloudsim.examples.network


org.cloudbus.cloudsim.examples.network.datacenter - package org.cloudbus.cloudsim.examples.network.datacenter


org.cloudbus.cloudsim.examples.power - package org.cloudbus.cloudsim.examples.power


org.cloudbus.cloudsim.examples.power.planetlab - package org.cloudbus.cloudsim.examples.power.planetlab


org.cloudbus.cloudsim.examples.power.random - package org.cloudbus.cloudsim.examples.power.random


org.cloudbus.cloudsim.lists - package org.cloudbus.cloudsim.lists


org.cloudbus.cloudsim.network - package org.cloudbus.cloudsim.network


org.cloudbus.cloudsim.network.datacenter - package org.cloudbus.cloudsim.network.datacenter


org.cloudbus.cloudsim.power - package org.cloudbus.cloudsim.power


org.cloudbus.cloudsim.power.lists - package org.cloudbus.cloudsim.power.lists


org.cloudbus.cloudsim.power.models - package org.cloudbus.cloudsim.power.models


org.cloudbus.cloudsim.provisioners - package org.cloudbus.cloudsim.provisioners


org.cloudbus.cloudsim.util - package org.cloudbus.cloudsim.util


org.workflowsim - package org.workflowsim


org.workflowsim.clustering - package org.workflowsim.clustering


org.workflowsim.clustering.balancing - package org.workflowsim.clustering.balancing


org.workflowsim.clustering.balancing.methods - package org.workflowsim.clustering.balancing.methods


org.workflowsim.clustering.balancing.metrics - package org.workflowsim.clustering.balancing.metrics


org.workflowsim.examples - package org.workflowsim.examples


org.workflowsim.failure - package org.workflowsim.failure


org.workflowsim.planning - package org.workflowsim.planning


org.workflowsim.reclustering - package org.workflowsim.reclustering


org.workflowsim.scheduler - package org.workflowsim.scheduler


org.workflowsim.utils - package org.workflowsim.utils


OTHER\_POLICY\_DIFFERENT\_RATING - Static variable in class org.cloudbus.cloudsim.DatacenterCharacteristics
:   Assuming all PEs in a Machine have the same rating.

OTHER\_POLICY\_SAME\_RATING - Static variable in class org.cloudbus.cloudsim.DatacenterCharacteristics
:   Assuming all PEs in all Machines have the same rating.

OUTPUT\_CSV - Static variable in class org.cloudbus.cloudsim.examples.power.Constants


OUTPUT\_SIZE - Static variable in class org.cloudbus.cloudsim.network.datacenter.NetworkConstants


OverheadParameters - Class in org.workflowsim.utils
:   This class includes all parameters that involve overheads

OverheadParameters(int, Map<Integer, Double>, Map<Integer, Double>, Map<Integer, Double>, Map<Integer, Double>, double) - Constructor for class org.workflowsim.utils.OverheadParameters
:   Created a new OverheadParameters object.

A B C D E F G H I J L M N O P Q R S T U V W Z

- Overview
- Package
- Class
- Use
- Tree
- Deprecated
- Index
- Help

- Prev Letter
- Next Letter

- Frames
- No Frames

- All Classes
